# MK2 Regulates Macrophage Chemokine Activity and Recruitment to Promote Colon Tumor Growth

**DOI:** 10.3389/fimmu.2018.01857

**Published:** 2018-09-21

**Authors:** Brandon B. Phinney, Anita L. Ray, Amanda S. Peretti, Stephanie J. Jerman, Carl Grim, Irina V. Pinchuk, Ellen J. Beswick

**Affiliations:** ^1^Department of Molecular Genetics and Microbiology, University of New Mexico Health Sciences Center, Albuquerque, NM, United States; ^2^Department of Internal Medicine, Division of Gastroenterology and Hepatology, University of Texas Medical Branch, Galveston, TX, United States

**Keywords:** MK2, macrophages, chemokines, inflammation, colon tumor

## Abstract

A major risk factor for colon cancer growth and progression is chronic inflammation. We have shown that the MAPK-activated protein kinase 2 (MK2) pathway is critical for colon tumor growth in colitis-associated and spontaneous colon cancer models. This pathway is known to regulate expression of the tumor-promoting cytokines, IL-1, IL-6, and TNF-α. However, little is known about the ability of MK2 to regulate chemokine production. This is the first study to demonstrate this pathway also regulates the chemokines, MCP-1, Mip-1α, and Mip-2α (MMM). We show that these chemokines induce tumor cell growth and invasion *in vitro* and that MK2 inhibition suppresses tumor cell production of chemokines and reverses the resulting pro-tumorigenic effects. Addition of MMM to colon tumors *in vivo* significantly enhances tumor growth in control tumors and restores tumor growth in the presence of MK2 inhibition. We also demonstrate that MK2 signaling is critical for chemokine expression and macrophage influx to the colon tumor microenvironment. MK2 signaling in macrophages was essential for inflammatory cytokine/chemokine production, whereas MK2^−/−^ macrophages or MK2 inhibition suppressed cytokine expression. We show that addition of bone marrow-derived macrophages to the tumor microenvironment enhances tumor growth in control tumors and restores tumor growth in tumors treated with MK2 inhibitors, while addition of MK2^−/−^ macrophages had no effect. This is the first study to demonstrate the critical role of the MK2 pathway in chemokine production, macrophage influx, macrophage function, and tumor growth.

## Introduction

Colorectal cancer (CRC) is one of the most common cancer types in both men and women (1). Inflammation increases the risk of developing CRC, as well as increasing the risk of mortality (2, 3). Advanced CRC, including tumors that do not respond to treatment, are associated with high levels of inflammatory cytokines (4, 5). Multiple inflammatory cytokines, including IL-1β, IL-6, and TNF-α, increase proliferation and invasion of tumor cells (6–8). We have previously shown that the MAPK activated protein kinase 2 (MK2) pathway is responsible for the majority of IL-1β, IL-6, and TNF-α production in both a colitis-associated cancer and a tumor transfer model of CRC (9, 10). MK2 is downstream of p38 MAPK. It is phosphorylated by p38 in the nucleus of cells and is transported to the cytoplasm where it stabilizes p38 (11). Perhaps the most well-known function of MK2 is post-transcriptional regulation of cytokines, as has been studied with IL-6 and TNF-α (12). However, there are several gaps in knowledge in how MK2 may regulate inflammation in the tumor microenvironment. The impact of this pathway on chemokines has not been well examined. Because cytokines and chemokines often regulate other cytokines/chemokines, a greater understanding of how MK2 signaling affects inflammation in the tumor microenvironment is needed.

Macrophages are an important source of tumor-promoting inflammatory cytokines. Although our previous work primarily focused on the essential role of the MK2 pathway in tumor cells for inflammatory cytokine production (10), here we show a potential role for MK2 in macrophages in the production of tumor-promoting cytokines. Only one study, to the best of our knowledge, shows the interaction of MK2 and MK3 signaling in macrophages and that MK2 is required for IL-10 production in an inflammatory setting (13). Here, we more closely examine the impact of this pathway in macrophages on colon cancer growth.

In the gut, recruitment of resident macrophages is dependent on MCP-1, Mip-1α, and Mip-2α (MMM) (14, 15). When inflammation occurs in response to injury or in the tumor microenvironment, gut-resident macrophages and other cells become activated and produce MMM. In turn, MMM promote macrophage migration and activation. Although the activity of these chemokines has mainly been examined for immune cell recruitment, MMM may also exhibit pro-tumorigenic activity. Studies in multiple cancers such as breast, prostate, and colon have shown a direct role for MCP-1 in increased proliferation of tumor cells (16, 17) and studies with MCP-1 receptor knockout mice or MCP-1 blockade have demonstrated a reduction in tumor size (18, 19). Furthermore, in gastric and bladder cancers, MCP-1 was shown to induce tumor cell EMT and invasion pathways (20, 21). Less is known about the direct effects of Mip-1α on tumors, although some studies suggest indirect tumor-promoting effects. Mip-2α may induce CRC tumor cell proliferation in a CXCR2-dependent manner (22) and also promote melanoma growth and metastasis (23). Although there is mixed information on these chemokines and tumor-promoting activity, the mechanisms of production and function during cancer progression remain unclear.

MK2 signaling is known to regulate IL-1β, IL-6, and TNF-α, but its impact on chemokines has not been examined. Because chemokines often regulate other cytokines, a greater understanding of how MK2 signaling affects inflammation in the tumor microenvironment is needed. For example, in one study, MCP-1 was shown to regulate IL-1β in a feedback loop (24) and since we showed that in colon cancer MK2 regulates the majority of IL-1β production in the colon tumor microenvironment (10), it is important to further study the downstream inflammatory effects of the MK2 pathway. Here, we examined the role of the MK2 pathway in the regulation of MMM in tumors. This is the first study to show that inhibition of MK2 leads to a dramatic decrease in production of these chemokines by both tumor cells and immune cells. Tumor cells treated with MK2 inhibitor demonstrated decreased macrophage influx, cytokine production, and tumor growth in animal models. Restoring MMM within tumors treated with MK2 inhibitors led to restoration of tumor growth. A similar effect occurred when wild-type bone marrow-derived macrophages were added to tumors. However, tumor growth was not restored when MK2^−/−^ macrophages were added to tumors. These results suggest an important unrecognized role of the MK2 pathway in macrophage recruitment and tumor-promoting chemokine production in CRC, and suggest MK2 should be further investigated as a therapeutic target.

## Materials and Methods

### Mice

Balb/c mice and C57/Bl6 mice were purchased from Envigo. MK2^−/−tm1Mgl^ mice were obtained from Dr. Mathias Gaestel, Hannover Medical University, and bred in a pathogen-free facility. All animal studies were approved by the University of New Mexico Health Sciences Center IACUC. Male and female mice were utilized at 6–10 weeks of age.

### CT26 Culture and Tumors

CT26 cells were purchased from ATCC (Manassas, VA, USA) and MC38 cells were obtained from NIH. Cells were cultured in complete RPMI with 10% FBS, 1% penicillin/streptomycin, and 1% l-glutamine. CT26 or MC38 cells were treated for 48 h before injection with either 50 μM of MK2 inhibitor PF364402 (Sigma Aldrich, St. Louis, MO, USA) or DMSO vehicle control. Cells were injected at 10^5^ for CT26 and 2 × 10^6^ for MC38 in 100 µl of PBS into the flank of 6- to 8-week-old Balb/c mice or C57/Bl6 mice. Some mice injected with cells exposed to MK2 inhibitors were injected intratumorally with recombinant MCP-1 (800 ng), Mip-1α (50 ng), and Mip-2α (700 ng) in 100 µl of PBS to replenish cytokine production or vehicle control. Treatments were administered 3 times/week from day 2 until day 13. Tumors were measured using calipers and divided for culture and RNA work.

### Multiplex Cytokine Arrays

Tumor tissue pieces were cut to 8 mg (±0.5 mg) and incubated for 16 h in complete medium. Tumor or cell culture supernatants were analyzed for cytokines by Luminex bead array (Millipore, Billerica, MA, USA) according to the manufacturer’s instructions.

### Proliferation and Invasion

For proliferation assays, 10^3^ cells were added to wells of 96-well plates. Cells were allowed to adhere for 4 h and then incubated with serum-free media as a baseline control or 10 ng/ml of MCP-1, Mip1-α, or Mip-2α for 4 days. Cells were then removed from plates with trypsin and counted using trypan blue to exclude dying cells. For invasion assays, tumor tissue was dissociated using 25 U/ml of collagenase I, II, and IV (Sigma Aldrich) incubated at 37°C and 5% CO_2_ for 20 min while rotating and run through the GentleMACS dissociator. Cells were filtered through a 40-µm filter, washed, counted, and plated in 96-well plates coated in recombinant fibronectin (R&D Systems, Minneapolis, MN, USA). Upon confluency, cells were serum starved overnight and a scratch created with a pipette tip. Matrigel (Corning Inc., Corning, NY, USA) was added to fill each scratch. Cells were incubated with MK2 inhibitor (50 µM PF-364402) or 10 ng/ml of MCP-1, Mip-1α, and/or Mip-2α. The scratch widths were measured immediately and 12 h later to calculate invasion through Matrigel.

### Western Blotting

Cells were treated for 30 min with 10 ng/ml of cytokines and lysed in RIPA buffer (Thermo Fisher Scientific, Rockford, IL, USA). Protein was quantified using BCA protein assay (Thermo Fisher Scientific). Protein (20 µg) was loaded into each lane of a 10% bis-acrylamide gel. The gel was electrophoresed at 120 V. Proteins were transferred to a membrane for 45 min at 100 V. The membrane was blocked overnight with 5% dry non-fat milk in TBS with 0.1% Tween-20. Phospo-MK2 antibody sc-101729 (Santa Cruz Biotechnology, Santa Cruz, CA, USA) was added at 1:1,000 for 2 h at room temperature. Rabbit anti-mouse-HRP (Thermo Fisher Scientific) was added at 1:10,000 for 1 h for 3A. For 3F, Sc-29313 for pMK2 at 1:500 with anti-mouse HRP (Biolegend) at 1:2000. Samples were visualized using SuperSignal West Femto substrate.

### Real-Time PCR

Tumor samples were homogenized in Ribozol (Amresco, Solon, OH, USA) and RNA extracted according to the manufacturer’s instructions. RNA was quantified with a Nanodrop (Thermo Fisher Scientific). The RT reaction mixture included random 2.5 µM hexamers, 500 µM dNTPs, 0.4 U/μl of RNase inhibitors, 5.5 mM MgCl_2_, MultiScribe Reverse Transcriptase (3.125 U/μl) and its buffer, and 1 µg of cellular RNA. The RT step was performed according to the following protocol: 10 min at 25°C, 60 min at 37°C, and 5 min at 95°C. The PCR reaction mix was prepared using the Assays-on-Demand™ gene expression assay mix (Applied Biosystems) for mouse β-actin, MCP-1, Mip-1α, Mip-2α, F4/80, and CD68 and 1 µl of cDNA was added to each PCR reaction mix. The reaction was carried out according to the following protocol: 2 min at 50°C, 10 min at 95°C (1 cycle), and 15 s at 95°C and 1 min at 60°C (45 cycles) on Applied Biosystem’s StepOnePlus instrument. The endpoint used in real-time PCR quantification, CT, was defined as the PCR cycle number that crossed the signal threshold. Quantification of cytokine gene expression was performed using the comparative CT method (Sequence Detector User Bulletin 2; Applied Biosystems) and reported as the fold change relative to the mRNA of the mouse housekeeping gene, β-actin.

### Confocal Microscopy

Frozen murine colon tumor tissue sections were fixed in 1% paraformaldehyde for 20 min at room temperature, blocked with normal rat serum (2.5% in PBS) and eFluor660-conjugated anti-murine F4/80 (0.2 µg/ml) rat mAbs (clone BM8) for 1 h at room temperature. The staining was followed by six washes with PBS with Ca^++^/Mg^++^. The sections were then mounted in SlowFade^®^ Gold antifade reagent with DAPI (Invitrogen, CA, USA). Confocal microscopy was performed with a Zeiss LSM880 laser-scanning confocal microscope (Carl Zeiss, Thornwood, NY, USA). To quantify F4/80 expression levels, six regions of each murine colon tumor section were measured for integrated density, area, and mean fluorescence with background readings from each image serving as control. The Corrected Total Cell Fluorescence [CTCF = Integrated Density − (area of selected image/mean background fluorescence)] was then calculated (25).

### Macrophage Culture and Adoptive Transfer

Bone marrow was collected from 6- to 8-week-old Balb/c, C57/Bl6, and MK2^−/−^ mice and cultured as previously described (26). For cytokine assays, 1 × 10^5^ macrophages were plated into 48-well plates overnight and exposed to vehicle control or 1 µg/ml of LPS (Sigma Aldrich) for 24 h for supernatant collection. Some cells were pre-treated with 50 µM of MK2 inhibitor overnight before LPS treatment. For adoptive transfer, 1 × 10^6^ macrophages were injected intratumorally on days 1 and 8.

### Statistics

Power analyses were performed to determine the sample size of the experimental and control groups to ensure that any effect is statistically detectable. An alpha of 0.05 was used, and the minimum acceptable power was 0.80. A minimum of five animals per group (to allow for experimental error) multiple independent experiments *in vivo* was used. Results were expressed as the mean ± SE. Differences between means were evaluated by one-way ANOVA in GraphPad Prism 5. Values of *p* < 0.05 were considered statistically significant.

## Results

### MK2 Inhibition Markedly Decreases MMM Production by Tumor Cells and Tumor Tissues

**Figure 1:**
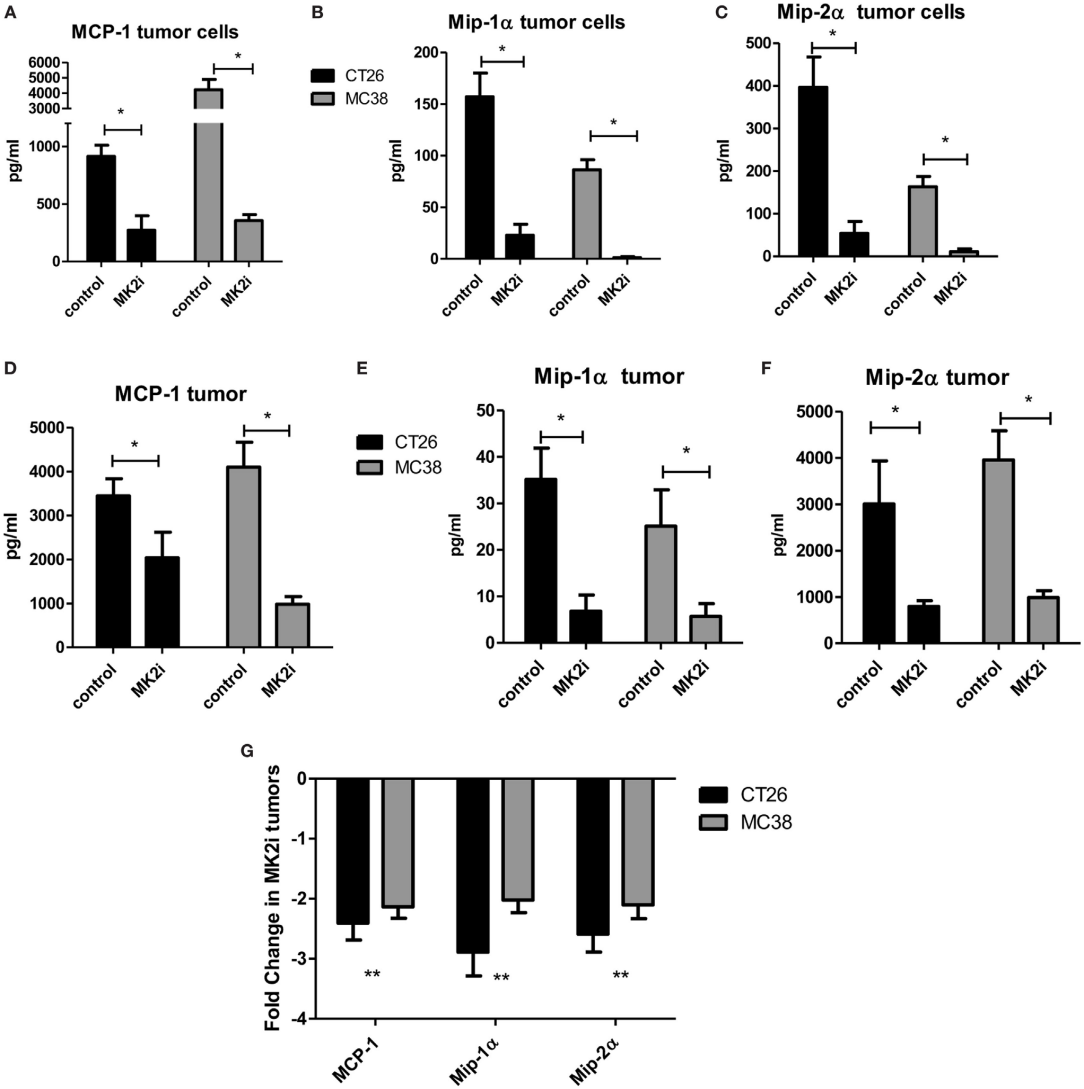
MK2 regulates colon cancer cell and colon tumor cytokine/chemokine production. CT26 and MC38 colorectal cancer cells in culture for 24 h produce **(A)** MCP-1, **(B)** Mip-1α, and **(C)** Mip-2α, which are drastically decreased upon MK2 inhibitor treatment (MK2i) as shown by multiplex bead array. Tumors developed from these cells in mice for 13 days show similar production of **(D)** MCP-1, **(E)** Mip-1α, and **(F)** Mip-2α when incubated overnight in media, which are also decreased with MK2i treatment. **(G)** MK2i-treated tumors also show a decrease in mRNA levels of these cytokines compared with control tumors. *N* = 6 for cultured tumor cells and 7–8 for tumors from multiple experiments, *p ≤0.05, **p ≤ 0.01, and ***p ≤0.001.

Our previous studies indicated that the MK2 pathway regulates IL-1β, IL-6, and TNF-α in CRC tumor models (9, 10). However, little is known about the role of MK2 signaling in the production of chemokines. Here, we expanded our studies on the impact of MK2 inhibition on tumor cell production of chemokines. CT26 and MC38 colon tumor cells in culture were found to produce MMM, which were decreased by up to 80% when cells were treated with MK2 inhibitor for 24 h in culture (Figures 1A–C). As we have previously reported, MK2 inhibition does not decrease viability of tumor cells (9, 10). To further examine the impact of MK2 inhibition on these chemokines *in vivo*, supernatants from control vs MK2 inhibitor-treated excised tumor tissue were examined at day 13. Multiplex array analysis revealed similar marked decreases in overall MMM from mouse tumor tissues when comparing the MK2 inhibitor group tumors to control group tumors (Figures 1D–F). Since cytokines and chemokines are thought to regulate each other, mRNA levels of MMM were also measured in tumors grown from control vs MK2 inhibitor-treated cells. The mRNA levels of all three chemokines were also decreased in tumors from the MK2 inhibitor groups by twofold to threefold compared with the control tumor group, which was similar in both CT26 and MC38 tumor groups (Figure 1G). Although regulation of chemokine production is complex, these results suggest that MK2 in involved in the regulation of CRC tumor production of MMM.

### MMM Induce Tumor Cell Proliferation, Invasion, and *In Vivo* Growth

**Figure 2:**
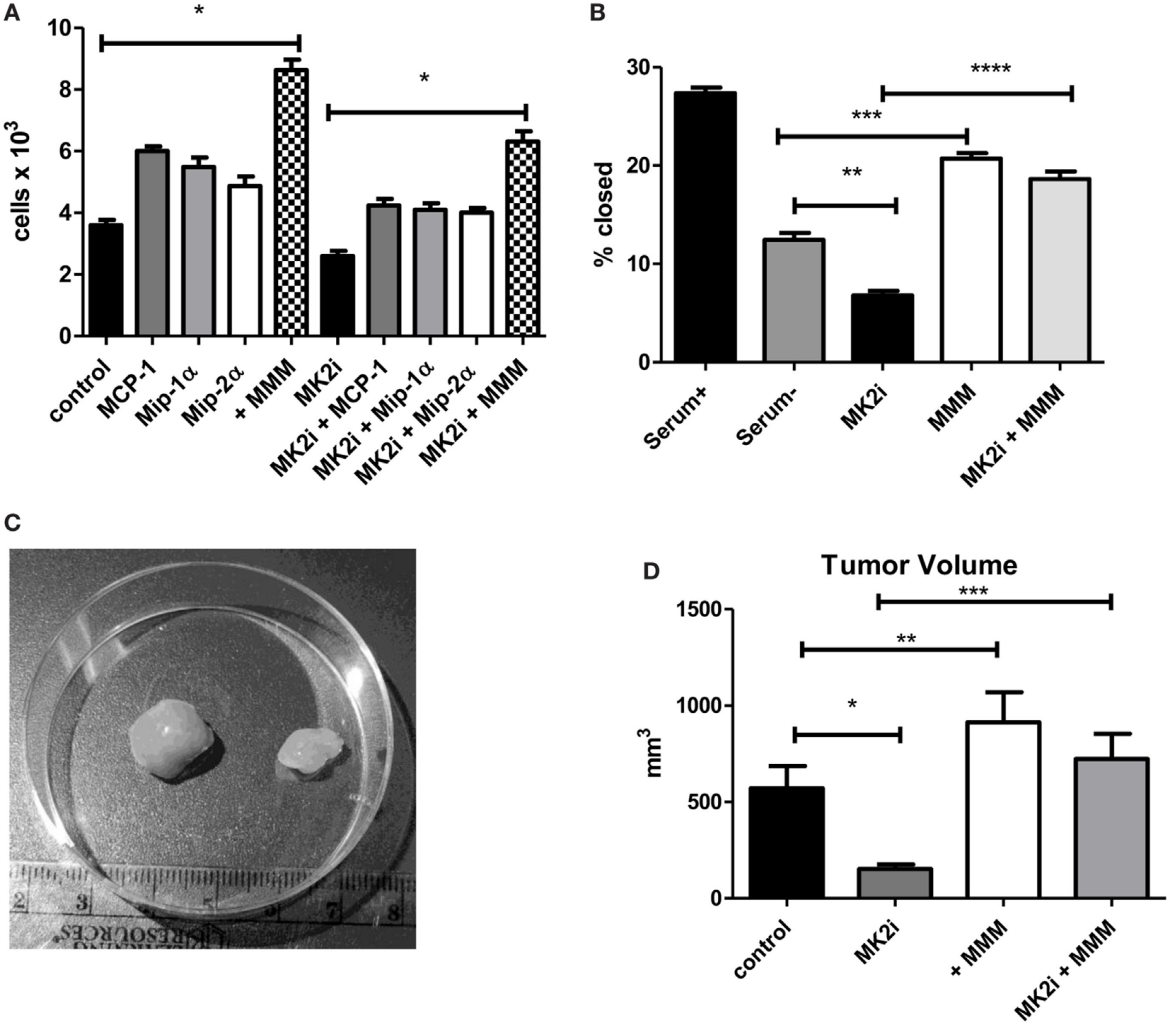
MCP-1, Mip-1α, and Mip-2 α induce tumor cell proliferation, invasion, and *in vivo* tumor growth in an MK2-dependent mechanism. CT26 cells in culture treated with MCP-1, Mip-1α, and Mip-2α (MMM) show increased **(A)** cell proliferation measured 4 days after treatment with chemokines and **(B)** invasion through Matrigel after 12 h, which is decreased upon MK2 inhibitor treatment. *In vivo*, **(C)** MK2 inhibition decreased tumor growth and **(D)** addition of MMM to tumors treated with MK2 inhibitor restores tumor growth by day 13. *N* = 8 for cultured tumor cells and 7–8 for tumors from multiple experiments, *, **, and ***p ≤ 0.05.

Tumor cells are known to express chemokine receptors and CT26 cells have been shown to express receptors for MMM (22, 27, 28). Thus, we examined the effects of these chemokines on tumor cell proliferation in culture. CT26 cells were exposed to MMM (10 ng/ml) and cell number counted after 4 days in culture. Cell numbers were significantly increased with each cytokine individually, but also showed a combined effect to enhance cell proliferation (Figure 2A). A similar result was observed with cells pre-treated with MK2 inhibitor where addition of these chemokines restored cell proliferation. Proliferation and invasion were also measured using a scratch-wound assay where Matrigel was added to the wound using an approach we have previously published (9, 10). The opening was measured at the beginning of the assay and 12 h later. MK2 inhibitor treatment decreased invasion, but addition of MMM increased invasion through the Matrigel to levels higher than the control (Figure 2B).

To examine the role of these chemokines in tumor growth *in vivo*, tumor growth from CT26 cells was examined. Cells treated with MK2 inhibitor led to substantially smaller tumors than control cells as shown in an example image at day 13 (Figure 2C). Addition of MMM to control tumors led to significantly increased tumor growth, while addition of the chemokines to tumors formed from MK2 inhibitor-treated cells restored tumor growth to control levels (Figure 2D) suggesting an important role in tumor growth for these chemokines. Taken together, these data suggest that activation of the MK2 pathway is involved in MMM production and these cytokines individually increase tumor cell proliferation, but in combination further enhance proliferation, invasion, and *in vivo* tumor growth.

### MMM Activate MK2 Signaling and Downstream Cytokine Expression in CRC

**Figure 3:**
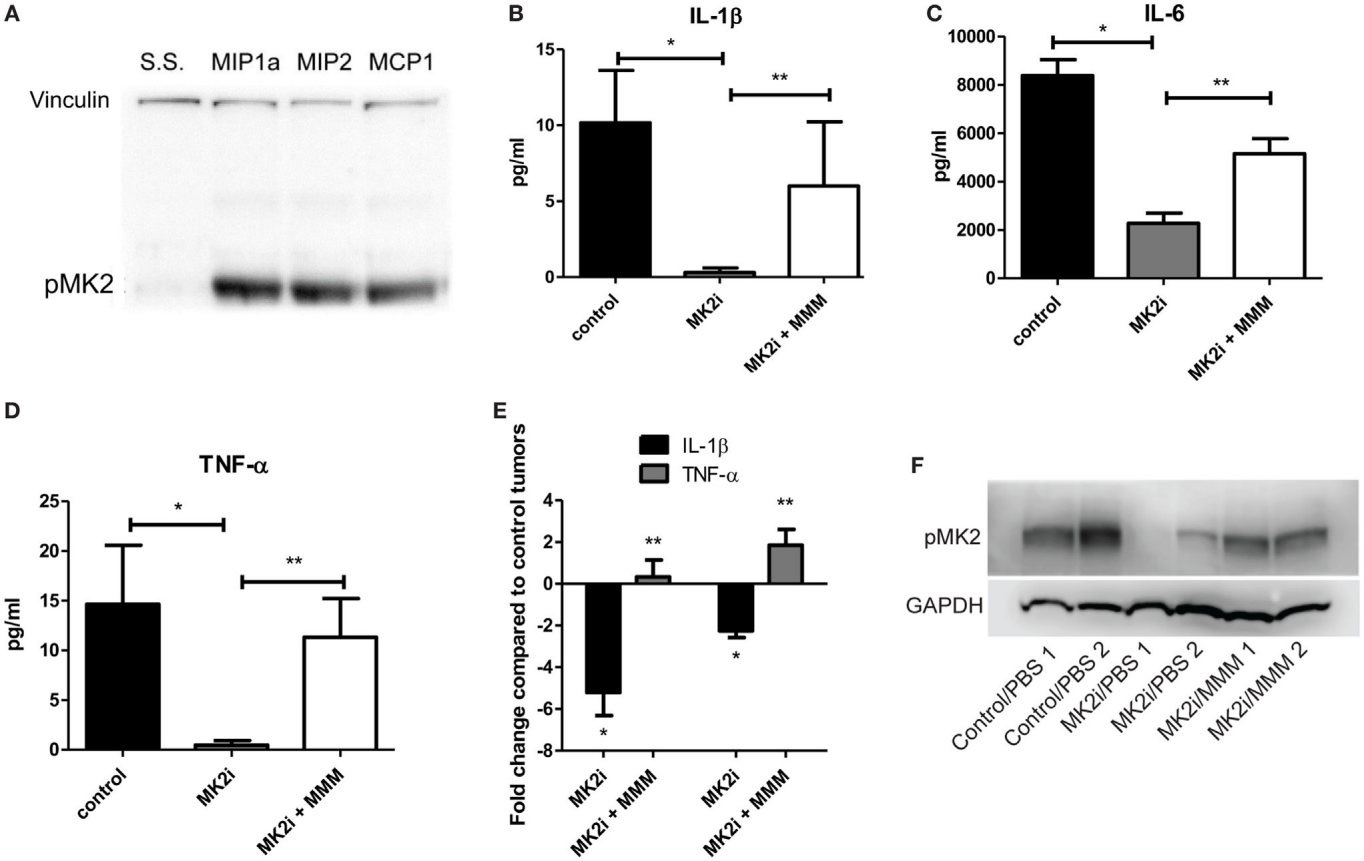
MCP-1, Mip-1α, and Mip-2α (MMM) activate MK2 signaling and downstream cytokine expression in colon cancer cells. CT26 cells treated with MMM induce **(A)** MK2 phosphorylation. MK2i treatment inhibits **(B)** IL-1β, **(C)** IL-6, and **(D)** TNF-α in tumor supernatants, but this effect is reversed upon addition of MMM when examined by multiplex cytokine array. **(E)** Additional mRNA data for IL-1β and TNF-α since the protein level is low support the increase in cytokine gene expression. **(F)** MK2 is also phosphorylated in CT26 tumors, is decreased at day 13 in tumors treated with MK2 inhibitor, and is restored by addition of MMM in tumor treated with MK2 inhibitors. *N* = 7–8 for tumors, * and **p ≤ 0.05.

Since we previously showed that cytokines activate MK2 in a feedback loop (10), the ability of MMM to activate MK2 was examined. CT26 cells were serum starved and exposed to 10 ng/ml of each chemokine for 30 min. All three chemokines induce MK2 phosphorylation (Figure 3A) suggesting a feedback loop for MK2 activation by downstream chemokines. Since MMM activated the MK2 pathway, the ability of these cytokines to induce IL-1β, IL-6, and TNF-α production was investigated. To further examine the mechanism of induction of these cytokines during CRC growth, supernatants from MK2 inhibitor-treated tumors were compared with supernatants from tumors that had addition of MMM. The known MK2 downstream cytokines, IL-1β, IL-6, and TNF-α were induced to a significantly higher level by addition of MMM (Figures 3B–E). The cytokine levels did not reach those of control tumors, which is not surprising since multiple pathways are known to regulate cytokine production. Tumors formed from CT26 cells also showed phosphorylated MK2, which continued to be decreased at day 13 (Figure 3F). However, addition of MMM to tumors treated with MK2 inhibitors restored MK2 phosphorylation suggesting autocrine and paracrine feedback mechanisms regulating MK2 activity. These results suggest a role for MMM in inducing MK2 signaling and promoting downstream cytokine production.

### Macrophage Influx Is Decreased in Tumors Treated With MK2 Inhibitor

**Figure 4:**
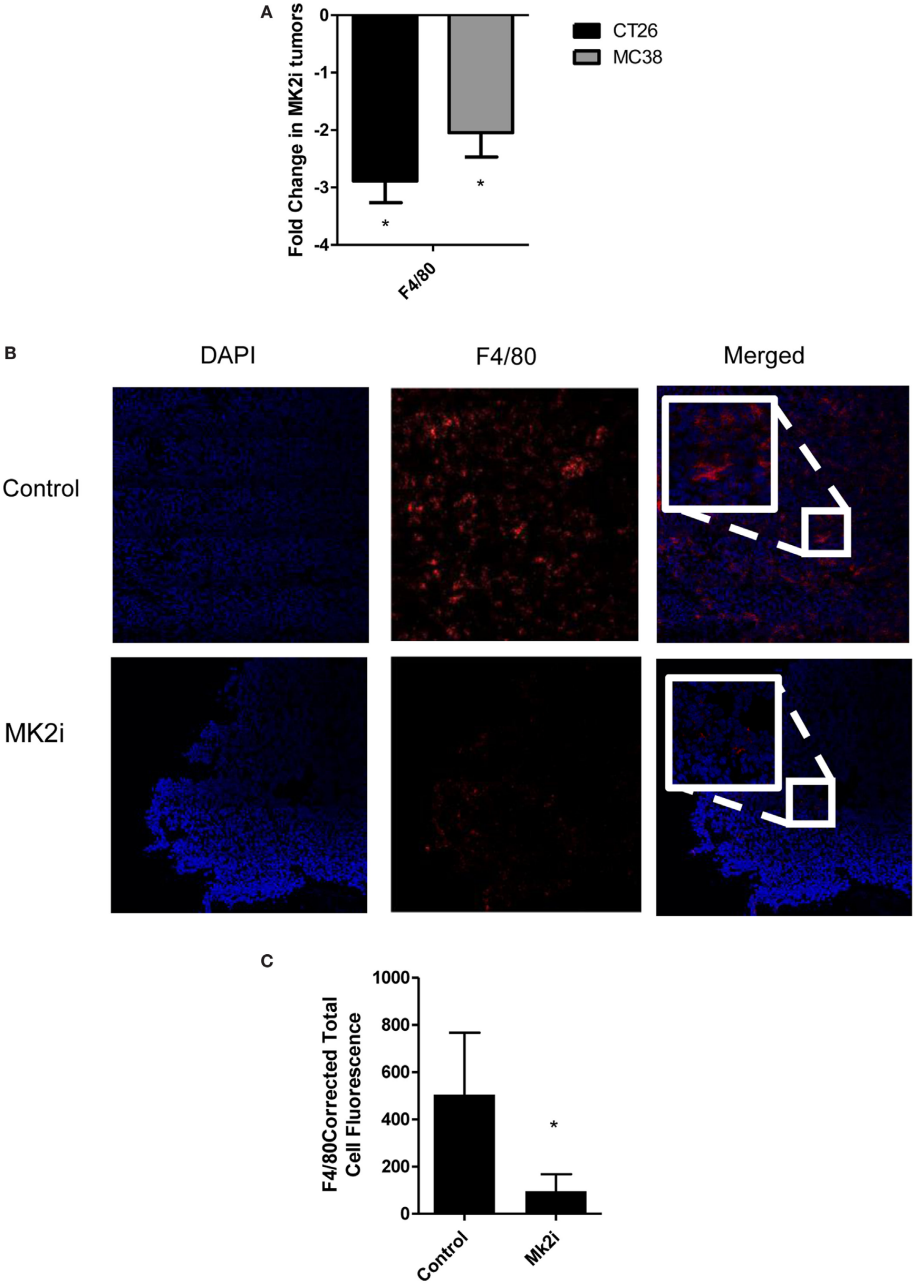
Macrophage influx is decreased in tumors treated with MK2 inhibitor. Tumors developed from cells treated with MK2 inhibitors showed decreased macrophage markers at day 13 by **(A)** mRNA levels of F4/80 and **(B)** by confocal microscopy of F4/80 staining with example images and **(C)** combined corrected total cell fluorescence from multiple images where the mean of 10 fields from each image was calculated divided by the mean background fluorescence. The squares show high power resolution view of a randomly selected area in each low power resolution image.

One of the major functions of MMM is chemotaxis of myeloid cells. Given that these chemokines are markedly reduced in MK2 inhibitor-treated tumors, macrophage markers in control and MK2i tumors were examined. First, mRNA levels of F4/80 were examined and approximately 2.5-fold decreased expression was seen in tumors treated with MK2 inhibitor compared with control tumors (Figure 4A). To confirm these results, F4/80 immunostaining followed by confocal microscopy was performed. F4/80 expression was measured in six regions of each colon tumor section. The F4/80 expression level was reduced in tumors treated with MK2 inhibitor by approximately fourfold (Figures 4B,C). These data suggest that MK2 activation is required for macrophage migration into tumors.

### The MK2 Pathway in Macrophages Is Critical for Cytokine/Chemokine Production

**Figure 5:**
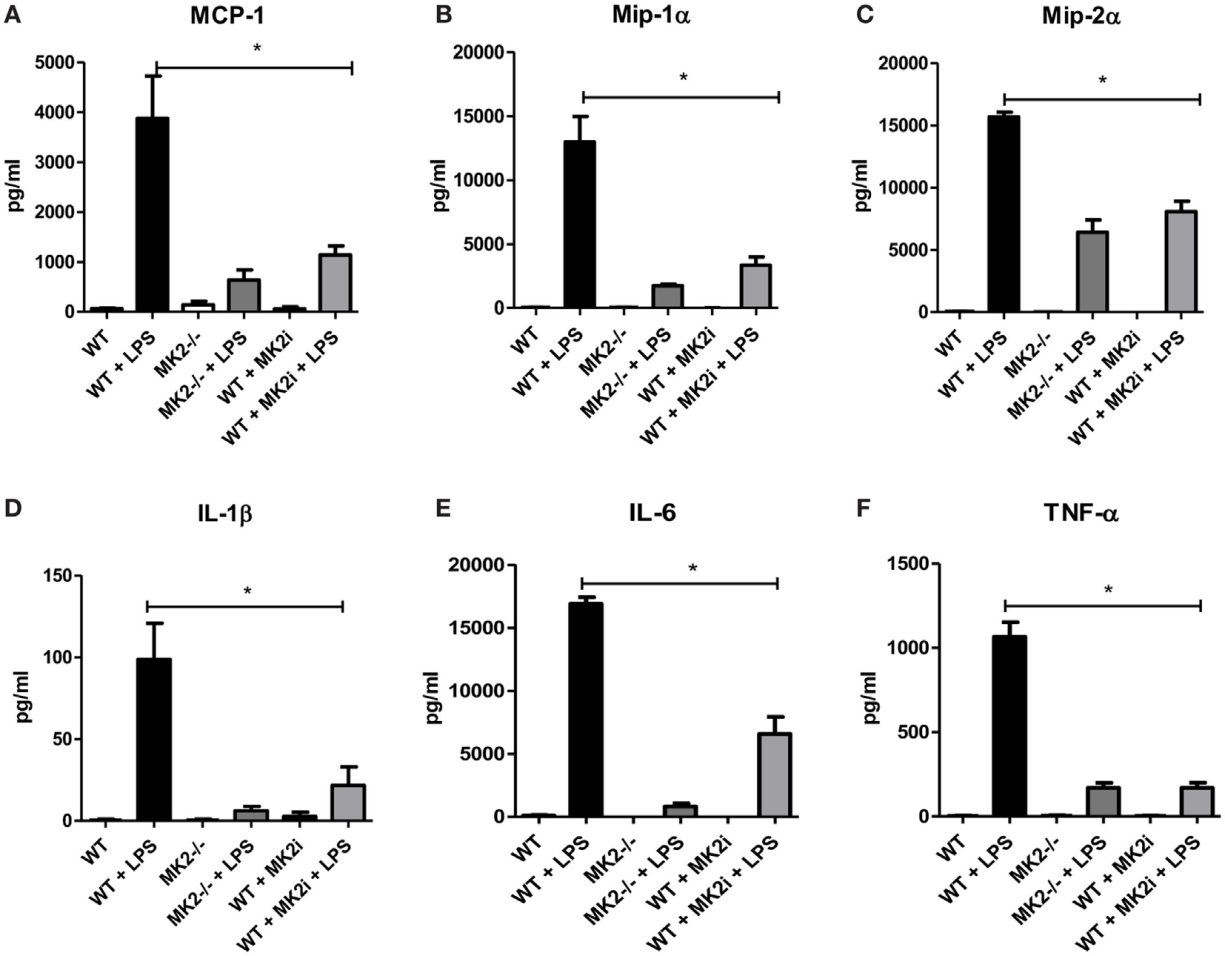
The MK2 pathway is critical for macrophage cytokine/chemokine production. Bone marrow-derived macrophages in culture were exposed to LPS for 24 h and cytokine production compared between WT, MK2^−/−^, and MK2 inhibitor-treated macrophages by multiplex cytokine array. WT macrophages showed increased production of **(A)** MCP-1, **(B)** Mip-1α, **(C)** Mip-2α, **(D)** IL-1β, **(E)** IL-6, and **(F)** TNF-α while MK2^−/−^ and MK2 inhibitor-treated macrophages had significantly decreased production. *N* = 8 from multiple experiments, *p ≤ 0.05.

Macrophages are thought to have pro- or anti-tumorigenic properties in the tumor microenvironment. Since we found macrophage chemokines and macrophages influx to be decreased in MK2i tumors, we sought to further investigate the role of the MK2 pathway in macrophage cytokine/chemokine production. Thus, WT and MK2^−/−^ bone marrow-derived macrophages were exposed to LPS to examine cytokine/chemokine production. WT macrophages were found to produce high levels of MMM, while MK2^−/−^ produced distinctly less (Figures 5A–C). Results were confirmed by pre-treating WT macrophages with MK2 inhibitor overnight before exposing cells to LPS. Furthermore, WT macrophages also produced high levels of the known MK2-regulated cytokines, IL-1β, IL-6, and TNF-α, while MK2^−/−^ and MK2 inhibitor-treated cells produced substantially less than WT (Figures 5D–F). These results suggest that MK2 regulates cytokine/chemokine production not only in tumor cells but also in macrophages.

### The MK2 Pathway in Macrophages Is Critical for CT26 and MC38 Tumor Growth

**Figure 6:**
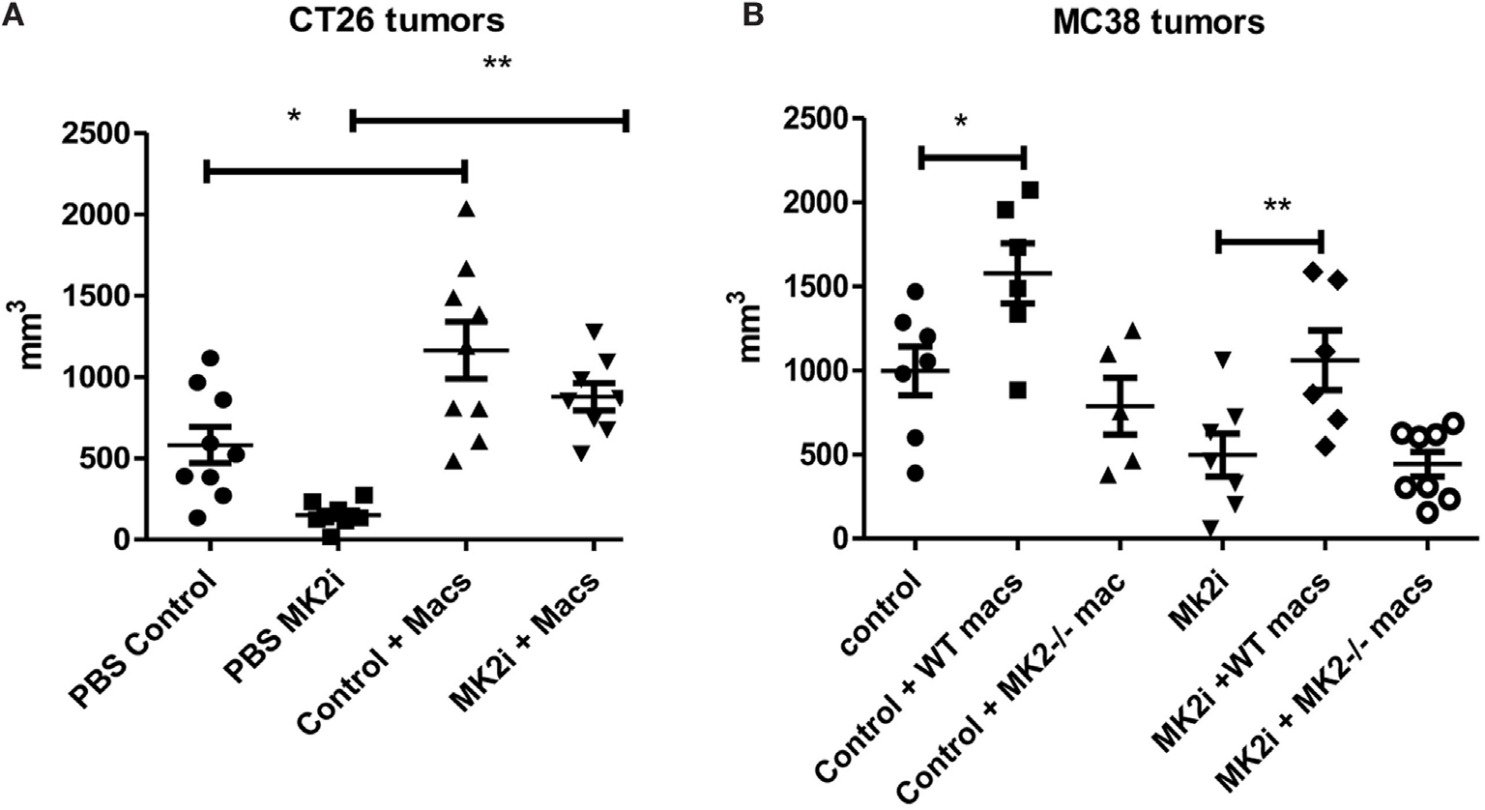
The MK2 pathway in macrophages is critical for CT26 and MC38 tumor growth. Mouse tumors grown from **(A)** CT26 cells were decreased in volume with MK2 inhibitor treatment, but increased with 10^6^ wild-type bone marrow-derived macrophage injection and **(B)** MC38 tumors were decreased in volume with MK2 inhibitor treatment, increased with additional wild-type macrophages, but not with MK2^−/−^ macrophage injection. All tumors were excised and measured at day 13. *N* = 7–8, * and **p ≤ 0.05.

Our group and others have shown that macrophages in the tumor microenvironment may promote tumor growth (9). Above, we showed that not only are macrophages decreased in MK2i tumors but also that MK2^−/−^ of WT macrophages treated with MK2 inhibitor secrete markedly decreased cytokines and chemokines. Thus, we investigated the impact of macrophages in the CT26 and MC38 tumors. Control or MK2i tumors were injected with 1 × 10^6^ bone marrow-derived macrophages on days 1 and 8 after tumor cell injection. Tumors were found to be significantly larger in both models with injection of macrophages (Figures 6A,B). In addition, while MK2i tumors were much smaller than control tumors, the addition of macrophages restored the tumor volume to that of control tumors.

Since the MC38 model is on the B6 background, for this model, we also injected MK2^−/−^ bone marrow-derived macrophages since MK2^−/−^ mice are on the same background. While WT macrophages enhanced tumor growth in both control and MK2i tumors, MK2^−/−^ macrophages did not, indicating the importance of the MK2 pathway in macrophages for promoting tumor growth (Figure 6B).

### Addition of Macrophages to MK2i Tumors Restores Cytokine and Chemokine Production

**Figure 7:**
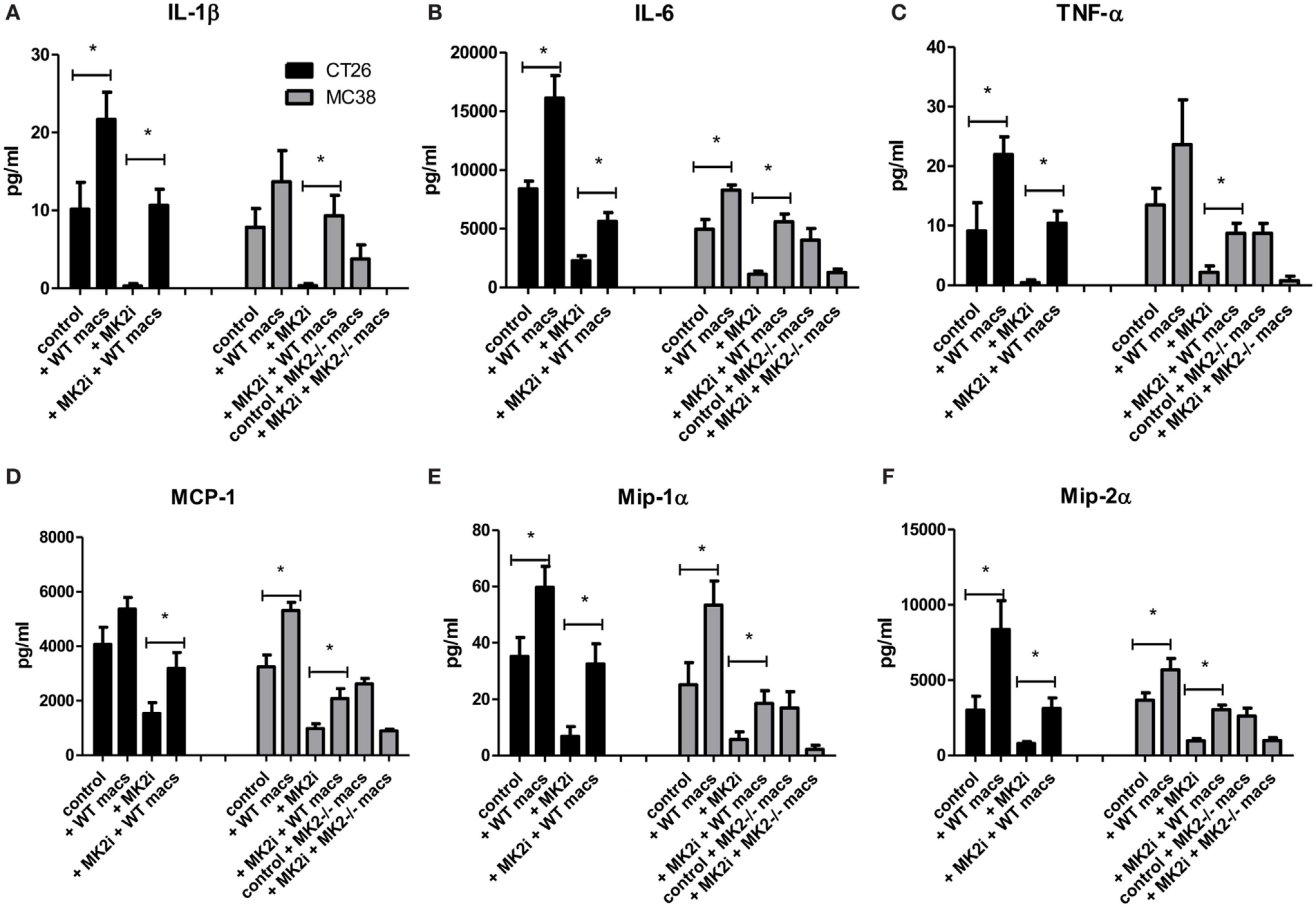
Addition of WT macrophages to MK2 inhibitor-treated tumors restores cytokine and chemokine production *in vivo*. CT26 and MC38 tumor supernatants produce **(A)** IL-1β, **(B)** IL-6, **(C)** TNF-α, **(D)** MCP-1, **(E)** Mip-1α, and **(F)** Mip-2α, which was decreased with MK2 inhibition and restored by the addition of macrophages to tumors by multiplex cytokine array. *N* = 7–8, *p ≤ 0.05.

Since decreased macrophage numbers and cytokines/chemokines were found in tumors upon MK2 inhibition, but restored upon addition of macrophages, the levels of cytokines and chemokines were examined in tumor supernatants. In both CT26 and MC38 models of colon cancer, addition of bone marrow-derived macrophages to tumors led to increased IL-1β, IL-6, and TNF-α at significant levels with the exception of IL-1β in MC38 tumors (Figures 7A–C). MK2i tumors also had an increase in these cytokines, with levels similar to control tumors for IL-1β and TNF-α, and slightly less than control for IL-6. MMM were also increased with adoptive transfer of macrophages in both models, all reaching significance with the exception of MCP-1 in the CT26 model (Figures 7D–F). For MK2i tumors, chemokine levels were replenished significantly, to approximately control levels for Mip-1α and Mip-2α, but not MCP-1. In contrast, in the MC38 model, addition of MK2^−/−^ macrophages did not increase cytokine and chemokine levels indicating the importance of this pathway in macrophages for the activation of tumor-promoting inflammatory responses.

## Discussion

There is a growing body of evidence that the MK2 pathway is a critical regulator of inflammatory cytokines. Although the most commonly studied cytokines regulated by MK2 are IL-1β, IL-6, and TNF-α, here we showed that MMM are also substantially decreased in tumor cells and in tumors with mixed cell populations upon MK2 inhibition. We found these cytokines/chemokines to be produced by tumor cells and to feedback on tumor cells to induce proliferation, suggesting that both cytokines and chemokines play a role in MK2-induced tumor cell proliferation and tumor growth in mice. We also show that MMM induce MK2 phosphorylation in colon cancer tumor cells suggesting an autocrine regulation mechanism. Few studies have focused on production of these chemokines in CRC or their tumor-promoting functions, but here we show that they have previously unrecognized tumor-promoting properties.

MCP-1 has been shown to have both pro- and anti-tumorigenic effects in both mouse and human tumors. Pro-tumor effects include tumor cell proliferation, migration, and metastasis in various cancers, such as renal, prostate, and melanoma (29–31). Conversely, in melanoma, MCP-1 was also suggested to promote tumor elimination *via* monocyte recruitment and lymphocyte infiltration (32, 33). Less information is available on the impact of Mip1-α on tumors; however, one study suggests that Mip-1α secretion in the lung and mammary gland promote enhanced proliferation of tumor cells (34). Mip-2α was also shown to induce proliferation of tumor cells in a CXCR2-dependent manner in breast cancer (22). Since there is evidence of MCP-1 chemokines promoting cell proliferation, we investigated their impact on colon cancer cells and found that they did enhance both *in vitro* cell proliferation and *in vivo* tumor growth.

Our study also indicates that during colon tumor growth activation of the MK2 pathway is critical for production of these chemokines since inhibition of this pathway led to an 80% decrease in production. Our previous study with colitis-associated cancer showed that MK2^−/−^ mice produced very little MCP-1 (9), and another study of arteriogenesis also showed that MK2^−/−^ mice showed decreased MCP-1 production (35). One study of spinal injury in MK2^−/−^ mice also shows a decrease in Mip-1α (36). Herein, we are the first to show that MK2 inhibition decreases Mip-2α production. Furthermore, regulation of these chemokines in the CRC tumor microenvironment has not been well studied, particularly in association with MK2 activity, and this work suggests a new tumor-promoting chemokine mechanism that is dependent on MK2 signaling.

We also found that chemokines may have a dual function in the colon tumor microenvironment by promoting tumor cell proliferation and increasing chemotaxis of macrophages. With MK2 inhibition, the number of macrophages was also decreased in tumors. However, addition of bone marrow-derived macrophages to tumors enhanced tumor growth. Others have also demonstrated that macrophages promote tumor growth, invasion, angiogenesis, and metastasis (37). These chemokines may affect chemotaxis of other immune cells to the tumor microenvironment that should be examined in future work. For example, Mip-2α is known to affect neutrophil chemotaxis, but it is not clear how neutrophils affect tumor growth in this model. There also may be a role for myeloid-derived suppressor cells in promoting tumor growth that should be further examined in this model.

*In vitro*, we found macrophages to produce MMM along with the known MK2-regulated cytokines IL-1β, IL-6, and TNF-α in an MK2-dependent manner. We previously reviewed that the MK2 pathway is important in macrophage function (38) and another group showed that MK2 plays an important role in LPS-mediated macrophage cytokine production (13). However, we are the first to show that when comparing tumor injection of WT and MK2^−/−^ macrophages, MK2^−/−^ macrophages did not enhance tumor growth as WT did. This is the first suggestion that the MK2 pathway is a critical driver of macrophage function in the tumor microenvironment. Recently, one group demonstrated that loss of MK2 in dendritic cells led to improved antitumor immunity (39) implying that further investigation is needed into the role of MK2 in regulating immune responses in the tumor microenvironment.

In conclusion, our study shows that MK2 activity in colon tumors and colon tumor-associated macrophages is a critical regulator of not only IL-1β, IL-6, and TNF-α but also MMM, which has not previously been reported. For the first time, we show that MMM induce MK2 phosphorylation in colon tumor cells and when added to tumors treated with MK2 inhibitor induce production of the known MK2-downstream cytokines IL-1β, IL-6, and TNF-α. The chemokines in this study and the cytokines in our previous study regulated by MK2 activity promote colon tumor cell proliferation and *in vivo* tumor growth. Finally, these studies demonstrate that MK2 regulation of cytokines and chemokines in the tumor microenvironment is complex, involves both tumor cells and immune cells, and promotes tumor growth by multiple autocrine and paracrine mechanisms.

## Ethics Statement

These studies were approved by the University of New Mexico Health Sciences Center Institutional Animal Care and Use Committee.

## Author Contributions

BP—study design and analysis, writing. AR—study design and data collection, review of manuscript. AP—data collection, review of manuscript. SJ—data collection and analysis. CG—data collection and analysis. IP—study design, interpretation of data, critical review of manuscript for intellectual content. EB—study design, interpretation of data, critical review of manuscript for intellectual content, writing, obtained funding.

## Conflict of Interest Statement

The authors declare that the research was conducted in the absence of any commercial or financial relationships that could be construed as a potential conflict of interest.
